# Vocal individuality cues in the African penguin (*Spheniscus demersus*): a source-filter theory approach

**DOI:** 10.1038/srep17255

**Published:** 2015-11-25

**Authors:** Livio Favaro, Marco Gamba, Chiara Alfieri, Daniela Pessani, Alan G. McElligott

**Affiliations:** 1 Department of Life Sciences and Systems Biology, University of Turin, Via Accademia Albertina 13, 10123 Turin, Italy; 2 Biological and Experimental Psychology, School of Biological and Chemical Sciences, Queen Mary University of London, Mile End Road, London E1 4NS, UK

## Abstract

The African penguin is a nesting seabird endemic to southern Africa. In penguins of the genus *Spheniscus* vocalisations are important for social recognition. However, it is not clear which acoustic features of calls can encode individual identity information. We recorded contact calls and ecstatic display songs of 12 adult birds from a captive colony. For each vocalisation, we measured 31 spectral and temporal acoustic parameters related to both source and filter components of calls. For each parameter, we calculated the Potential of Individual Coding (PIC). The acoustic parameters showing PIC ≥ 1.1 were used to perform a stepwise cross-validated discriminant function analysis (DFA). The DFA correctly classified 66.1% of the contact calls and 62.5% of display songs to the correct individual. The DFA also resulted in the further selection of 10 acoustic features for contact calls and 9 for display songs that were important for vocal individuality. Our results suggest that studying the anatomical constraints that influence nesting penguin vocalisations from a source-filter perspective, can lead to a much better understanding of the acoustic cues of individuality contained in their calls. This approach could be further extended to study and understand vocal communication in other bird species.

Animal vocalisations have the potential to provide a variety of information about age, body size, sex, social status, and behavioural state of the emitter^1^,^2^. Vocalisations are also a prominent channel for signalling individual identity to conspecifics^3^,^4^,^5^,^6^,^7^,^8^. Discriminating among individuals is important for almost all social behaviours^9^ and the evolution of vocal individuality has been shown to be related to the size of social groups^10^. Accordingly, species living in larger groups have more signature information in their calls compared to species that live in smaller (even if more complex), social units^10^. In birds, evidence for individual acoustic variation in vocal signals exists in a wide range of species^11^ and acoustic features of vocalisations are shaped by genetic, developmental, and environmental factors^12^. Accordingly, mechanisms to encode the acoustic cues of individuality, range from amplitude^13^,^14^ and frequency modulations^15^,^16^ to the sequence of vocal units in songbirds^17^,^18^.

The source-filter theory of vocal production^19^ is a robust framework for studying individuality cues in the vocalisations of many non-human mammals^20^, where acoustic variation can originate from individual distinctiveness in the morphology or size of the vocal apparatus. More recently, the source-filter theory has emerged as the dominant theory for also explaining the acoustic output of many bird vocalisations^21^,^22^,^23^,^24^,^25^. According to this theory, bird calls are produced by the syrinx (the source), a two-part vocal organ located at the base of the trachea^26^,^27^, through vibrations of membranes (determining the fundamental frequency, “*f*_0_”). Subsequently, the sound passes through the suprasyringeal vocal tract (filter, formed by the tracheal tube, larynx, glottis (corresponding to the opening of the larynx), oropharyngeal cavity and beak. When the signal produced at the syrinx passes through the vocal tract, its frequency spectrum is shaped by the frequency selectivity of the suprasyringeal cavities^28^. The prominent peaks, corresponding to frequency bands whose energy has been left unchanged or slightly increased, are called “formants”. Individual variation in length and shape of the vocal apparatus can result in individual distinctiveness in the *f*_0_ and, above all, formants^6^,^28^,^29^. There is growing evidence that several bird species are perceptually sensitive to formants variation in vocalisations^30^,^31^.

Penguin vocalisations are highly exposed to ecological sources of selection, because these birds live in extreme climate conditions vocal signals have to propagate through high levels of background noise. Moreover, mechanisms for vocal recognition vary among species and according to their breeding ecology^32^,^33^. In particular, penguins of the genus *Aptenodytes*, which breed in extreme weather conditions on the Antarctic or sub-Antarctic islands, incubate their eggs on their feet to prevent them from freezing. Both the Emperor penguin (*Aptenodytes forsteri*), which does not have a nesting site or a meeting point for partners to reunite after foraging at sea, and the King penguin (*Aptenodytes patagonicus*), which have to identify particular areas of the colony where the partner is incubating, evolved finely tuned vocal recognition systems. They can localise and recognise a parent or a mate in huge crowded colonies of thousands of individuals, where vocalisations have a very low signal-to-noise ratio^34^,^35^,^36^,^37^. In particular, since the syrinx of birds is a two-part organ, where two independent sounds can be simultaneously generated by different sets of muscles and membranes at the right and left sides, non-nesting penguins have evolved the ability to use the beats generated by these different signals (the two-voice system), together with temporal characteristics of calls (variations in frequency or amplitude with time), to recognise each other. By contrast, the identification of the caller in penguins that build a nest (e.g. Adélie penguin, *Pygoscelis adeliae*, Gentoo penguin, *Pygoscelis papua, or* Macaroni penguin*, Eudyptes chrysolophus)* mostly occurs with relatively simpler mechanisms. In particular, they first locate their partner or chicks at the nest and then use vocalisations as a confirmation of the location cue. Mechanisms for vocal individuality in these species include frequency modulation of the pitch or the relative energy content of the harmonics^32^,^38^.

Penguins are a monophyletic family (Spheniscidae) of seabirds that is divided into six different genera and 18 extant species^39^. All the species live exclusively in the Southern Hemisphere, mostly concentrated in cooler waters. However, their distribution extends to southern Africa and South America, because of the Benguela and Humboldt currents, respectively^40^. Banded penguins form the genus *Spheniscus*. This genus comprises four extant species^41^ that inhabit temperate and equatorial areas of Southern Africa (African penguin, *S. demersus*), South America (Magellanic penguin, *S. magellanicus* and Humboldt penguin, *S. humboldti*) and the Galápagos Archipelago (Galápagos penguin, *S. mendiculs*). These species have similar body sizes^33^ and share common morphological traits. Sexual dimorphism is not evident but males are slightly larger than females. Moreover, banded penguins share similar behavioural ecology and nesting behaviours^42^. In particular, all species build nests in underground burrows that they excavate themselves^43^.

Nesting *Spheniscus* penguins can potentially recognise each other from vocalisations. Jouventin^44^ already reported that to the human ear, intra-individual similarities and inter-individual differences were apparent in the ecstatic display song of the African penguin. The song is composed of three acoustically distinctive syllables (Fig. 1), uttered both during exhalation (Types 1 and 2) and inhalation (Type 3) of air, which are combined in a phrase (for more detail see Favaro *et al.*^45^). Thumser and Ficken^46^ showed the presence of individual variation in number of syllables, duration of the longest syllable, and main frequency among different captive African penguins. In the same study, they also found individual variation in inter-syllable intervals and maximum frequency of the longest syllable in Magellanic penguin. Finally, they suggested the presence of individual distinctiveness in duration, minimum and maximum fundamental frequency in the contact calls of the Humboldt penguin. However, individual variation was not tested for contact calls in the other species due to the limited number of acoustic recordings available. More recently, Clark *et al.*^47^ demonstrated (using playbacks experiments of calls), that Magellanic penguins actually show partner recognition based on their vocalisations. In particular, females respond more strongly to ecstatic display songs of mates versus neighbours and strangers. Moreover, Clark *et al.*^47^ observed a significantly stronger response to penguins’ own mutual display songs than to that of stranger pairs. Finally, they reported that chicks are more responsive to the mutual display song of their parents compared to strangers. However, the study did not determine the acoustic features of calls that encode the individual identity information.

Recently, Favaro *et al.*^45^ provided a detailed description of the vocal repertoire of the African penguin. Favaro *et al.*^45^ showed that adult birds produce four basic vocalisations; namely, a contact call emitted by isolated birds, an agonistic call used in aggressive interactions, a mutual display song vocalised by pairs, at their nests, and an ecstatic display song uttered by single birds (almost exclusively males during the breeding season).

The goal of this study was to test whether African penguin vocalisations have the potential to provide information about the individual identity of callers, and to determine which parameters of contact calls and the ecstatic display song are responsible for individual identity. Lastly, we determined if the application of source-filter theory of vocal production could lead to a better understanding of the acoustic cues of individuality contained in vocalisations of this species. If shown to be reliable, the source-filter approach could be adopted to study and understand vocal communication in other bird species.

## Results

### Contact calls

Descriptive statistics for all the acoustic parameters measured on contact calls are provided in Table 1. The Potential of Individual Coding (PIC) value was ≥1.1 for 18 acoustic parameters, across the 24 that were measured. Using these parameters as independent variables, the discriminant function analysis (DFA) correctly classified 76.4% of the calls to the six individuals. The accuracy of the DFA decreased to 66.1% when the more conservative leave-one-out cross-validation was applied. The statistical significance of this classification for each individual and across individuals is presented in Supplementary Table S1. In addition, the stepwise analysis was performed in 10 steps and resulted in the further selection of 10 acoustic parameters important for vocal distinctiveness. These included five source-related (*f*_0_Mean, *f*_0_Max, *f*_0_AbsSlope, Jitter, Shimmer) and three filter-related (F_1_Mean, F_2_Mean, VTLest) measures, the harmonic to noise ratio (Sonority) and the duration of the call (Dur).

### Ecstatic display songs

Visual examination of the spectrograms showed that the ecstatic display song of the African penguin has considerable intra-individual stereotypy (Fig. 2). Descriptive statistics of acoustic parameters are provided in Table 2. The PIC value was ≥1.1 for 14 acoustic parameters across the 31 measured. Using these parameters as independent variables, the discriminant function analysis (DFA) correctly classified 71.9% of the ecstatic display songs for the seven individual penguins. When applying a leave-one-out cross-validated DFA this value dropped to 62.5%. The statistical significance of the DFA classification for each single bird and across individuals is presented in Supplementary Table S1. Moreover, the stepwise analysis was performed in 11 steps, and resulted in the further selection of nine acoustic parameters important for vocal distinctiveness. These included five source- (*f*_0_Start, *f*_0_Mean, *f*_0_Min, FMExtent) and one filter- (F_1_Mean) related measures, and four parameters related to number and duration of the different syllable types (DurType2, Type2, ∑Type2, ∑Type3).

## Discussion

Individual recognition is considered to be essential for animal sociality^9^,^10^. This explains why individually distinctive vocal features have been found in many social birds and mammals^3^,^7^,^8^,^9^,^10^,^11^,^33^,^48^,^49^. However, mechanisms used by animals to encode the vocal identity information are usually species-specific and are shaped by different genetic, developmental and environmental pressures^4^,^33^,^35^,^50^,^51^. We investigated the potential indicators of individuality in the contacts calls and ecstatic display songs in a territorial and colonial flightless seabird, the African penguin. We found evidence that 18 acoustic parameters for the contact calls and 14 for ecstatic display songs have low within-individual variation and high between-individual variation. In penguins, the ability to identify conspecifics using vocal cues is required for almost all social behaviours. For example, locating other birds using contact calls is needed to maintain cohesion when individuals are visually separated from the group or from particular social partners when foraging at sea^44^. In addition, vocal individuality is used for mate choice^52^ and parent-offspring recognition^34^,^35^,^47^. The study of vocal individuality in seabirds can contribute to our understanding of the evolution of their complex vocal communication systems, and how these have been shaped under different ecological pressures^53^. Furthermore, these data can be used to determine when and why the mechanisms used to encode information in vocalisations have diverged across species^32^,^37^,^38^,^53^,^54^.

**Figure 1:**
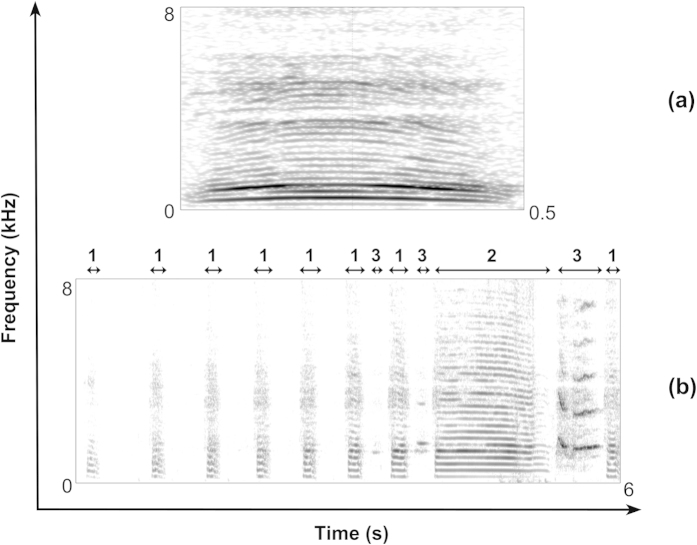
Spectrographic representation of contact call (a) and the ecstatic display song (b) arrows indicate short initial syllables 1, longest syllable 2, and inspiration syllable 3). Spectrograms were generated in Praat v. 5.4.01 sound editor window (Gaussian window shape, view range = 0 to 8000 Hz, window length = 0.02 s, dynamic range = 50 dB).

**Figure 2:**
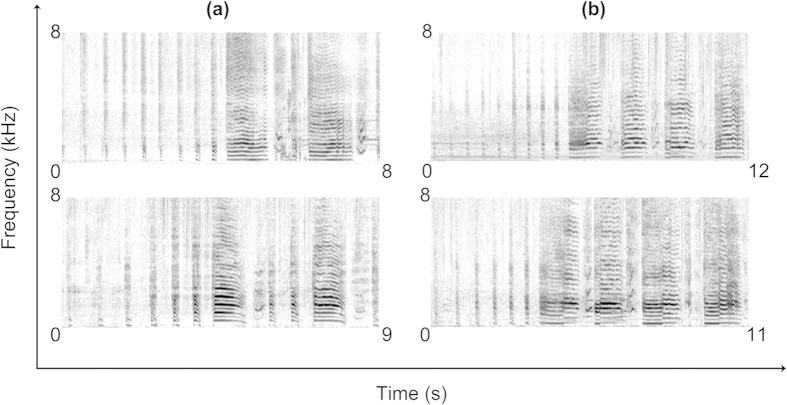
Ecstatic display songs recorded from two penguins (a,b) over different days provided as an example of the intra-individual stereotypy of the vocal units across the song. Spectrograms were generated in Praat v. 5.4.01 sound editor window (Gaussian window shape, view range = 0 to 8000 Hz, window length = 0.02 s, dynamic range = 50 dB).

Our results demonstrate how the source-filter theory of vocal production^19^ can be used to gain a better understanding of the biologically meaningful information contained in calls of nesting penguins. In particular, we showed that vocalisations in the African penguin can be studied by considering independent contributions from three different parts of the respiratory apparatus: lungs (temporal patterns), vocal production organ (source) and vocal tract (filter). We suggest that each of these three main motor systems contribute to encoding the individual identity in vocalisations. The chest muscles and lungs regulate exhalation, which determine the duration of contact calls and the temporal patterns in the ecstatic display song. The most prominent temporal features include the number and duration of inhalation (syllable type 3) and exhalation (syllable types 1 and 2) phases as well as the inter-syllable intervals (Fig. 1). The vocal organ (syrinx) transforms the airflow from lungs into acoustic energy. In particular, the vibration of the syringeal membranes controls the pitch of the call. Finally, the vocal tract has resonant cavities that change in volume and shape across individuals and generate amplified frequency bands, namely formants^19^,^20^. Formants alter the spectral structure of the sound and the distribution of the energy across the spectrum, which can, therefore, vary according to individual morphological distinctiveness.

The combination of the source and filter systems in birds can shape vocalizations in strikingly different ways^55^. Some species (e.g. doves) show a static filter that is used to amplify the fundamental frequency of a voice source not modulated beyond the formant bandwidth. Other species have a dynamic filter tracking fundamental frequency modulations during phonation (e.g. many songbirds). The last case is the dynamic filter with many independent bands of modulations, similarly to the event that we showed for the African penguin. Moreover, we observed stable and flat formants in both contact calls and ecstatic display songs. A stable vocal tract configuration during phonation usually results in stable formants, which have been recently suggested to make vocalizations particularly suitable for individual recognition^56^. In particular, the very stereotyped calling posture^45^,^57^ and the formant patterns we observed suggest that African penguins do not remarkably change the length or shape of their vocal tract during vocal production. Overall, our findings add important information to a growing body of literature on the importance of source- and filter-related acoustic cues in animal vocalisations. In particular, we supported its emerging role to explain the acoustic output of avian vocalisations^21^,^22^,^24^,^25^,^55^.

Our results provide evidence that contact calls can be used to advertise identity in penguins. Contact calls are vocalisations mostly used by birds and mammals^58^, which likely have evolved as social signals to maintain cohesion in stable groups^59^. However, there is growing evidence that contact calls can also be used for individual recognition^60^, which is particularly important in fission-fusion societies^4^. When we examined the role of the different vocal features to individual discrimination, DFA showed an accuracy of 66.1% for contact calls in the cross-validated procedure. Source-related (five parameters) and filter-related (three) parameters contributed most to the individuality, with some additional contributions by the duration of calls and the harmonic-to-noise ratio. These findings confirm that pitch and energy distribution across the spectrum of calls are both useful pathways to convey individual identity of nesting penguins^33^,^37^. Previous studies showed that the African penguin uses the contact call to maintain group cohesion when visually isolated from conspecifics^45^ or partners^46^ and especially when foraging at sea^44^. In captive settings, juveniles swimming alone in ponds may also emit contact calls^46^. Further research, investigating whether contact calls might allow also sex, mate, and kin recognition in this group of seabirds would be especially valuable.

Our results confirm that the ecstatic display song of *Spheniscus* penguins is composed of three acoustically distinct type of syllables arranged to form a sequence^45^,^46^ (Fig. 1). Our findings also showed that this vocalisation encodes individual identity information. However, the DFA performed to classify the ecstatic display songs showed an accuracy of 62.5%. This is lower accuracy than that obtained (100%) by Robisson *et al.*^61^ using 58 display songs from seven adult male Emperor penguins (*Aptenodytes forsteri*). In addition, the PIC values measured on the acoustic parameters of Emperor penguin vocalisations were higher than those we obtained for the ecstatic display songs of African penguins. We therefore find support for the hypothesis that the individual identity information encoded in non-nesting penguins is stronger than in species that build a nest^33^,^37^,^62^.

For the classification of the ecstatic display songs according to the emitter, the number and mean duration of the syllables type 2 and the relative contribution of each syllable type to the total duration of the song both contributed to the correct assignment of vocalisations. Moreover, in the stepwise procedure, the DFA used four source-related acoustic parameters and the mean value of the first formant to distinguish among individuals. The results of the DFA confirm that the ecstatic display song of the African penguin contains identity information in both temporal and spectral domains. However, similarly to what has been observed by Searby *et al.*^38^ for the Macaroni penguin (*Eudyptes chrysolophus*), our findings suggest that the signature system of the African penguin is not determined by a limited number of highly discriminant acoustic variables. By contrast, individual identity information in display songs is spread among several less discriminant vocal features.

In conclusion, we determined which acoustic features of contact calls and display songs have the potential to encode the individual identity information in the African penguin. Moreover, we showed that the source-filter theory of vocal production can lead to a far better understanding of the biological meaningful information encoded in penguin calls. This approach could be further extended to study vocal communication in other bird species.

## Methods

### Ethics statement

The study complies with all applicable Italian laws and was conducted in accordance with the Guidelines for the Treatment of Animals in Behavioural Research and Teaching^63^. Penguins were recorded without performing any manipulations and without the use of playback stimuli. Since all recording procedures were non-invasive and did not cause any disturbance to the animals during their normal daily activity, this study does not fall in any of the categories for which approval of an ethic committee is required by Italian laws.

### Subjects and housing

The study was performed using 12 adult African penguins belonging to a captive colony of 59 individuals at the “Bolder Beach” enclosure of the biopark Zoom Torino (44.933356 N, 7.419773 E), Italy. The original colony was established in 2009 by combining 37 adult African penguins hatch in four different zoological facilities in Europe (Artis Royal Zoo, Amsterdam, NL; Bird Park Avifauna, Alphen an den Rijn, NL; Wilhelma Zoo, Stuttgart, DE; South Lake Wild Animal Park, Manchester, UK). The colony increased to it current population because of several pairs reproducing. The colony was housed in an outdoor exhibit (1,500 m^2^, including a pond of 120 m^2^, water depth maximum 3 m), which reproduces the habitat of “Boulders Beach,” a natural nesting site in South Africa. All penguins were habituated to the presence of visitors and to close observations for recording vocalisations and behavioural studies^45^,^64^. Additionally, all birds had a microchip transponder and a flipper band for individual identification.

### Acoustic recordings and selection of vocalisations

Contact calls (Supplementary Audio S1) and ecstatic display songs (Supplementary Video S1) (Fig. 1) were collected using the focal animal sampling method^65^ over 10 non-consecutive days during May 2014, and 40 non-consecutive days from September to November 2014 (corresponding to the peak of the breading seasons for the captive colony). Vocalisations were collected at a distance of between 2 and 5 m from the caller with a RØDE NTG2 condenser transducer microphone (frequency response 20 Hz to 20 kHz, max SPL 131 dB). In order to reduce recorded noise, the microphone was mounted on a RØDE PG2 Pistol Grip and protected with a windscreen. The microphone was connected to a TASCAM DR-680 digital recorder (44.1 kHz sampling rate) and acoustic data were saved into an internal SD memory card in WAV format (16-bit amplitude resolution). All the files were then transferred to a Macintosh computer for later acoustic analyses.

We analysed 100 hours of audio recordings. For each audio file, we used narrow-band spectrograms to visually inspect the overall spectral structure of vocalisations. In particular, the waveform and the FFT (Fast Fourier Transform) spectrogram were generated with the Praat v. 5.4.01 sound editor window, using a customised spectrogram setting [view range = 0 to 8000 Hz, window length = 0.02 s, dynamic range = 50 dB]. A total of 221 vocalisations were excluded because they had excessive background noise or because calls were overlapping between different penguins vocalising at the same time. Overall, the spectrographic selection left us with a total of 118 contact calls (contributed by 6 individuals) and 64 ecstatic display songs (contributed by 7 individuals) to be used for the acoustic analysis. Table 3 shows the contribution of each African penguin recorded.

### Acoustic analysis

For each vocalisation, we measured a series of spectral and temporal acoustic parameters (Table 4), which were potentially important to discriminate between individuals. These included both temporal measures, such as call duration (Dur), and intensity measures, related to lung capacity^66^, source-related vocal features (*f*_0_), and filter-related acoustic vocal features (formants)^19^,^20^,^55^. However, before getting into the measurements of filer- related acoustic parameters, we estimated the approximate vocal tract length (VTL) for African penguins, to set a plausible number of formants in a given frequency range. We built computational models of the penguin vocal tract deriving information from silicon casts (for details please refer to Gamba and Giacoma^67^; Gamba *et al.*^68^) of two cadavers kept frozen at −20 C°. These individuals (one male and one female) died from natural causes in 2011 and 2012, respectively. The penguins were observed emitting the calls with an open beak but we did not know how the air resonated in the suprasyringeal tubes. In this species, the trachea is divided by a septum for all its length^69^ and shows a single tube only in the upper portion of the vocal tract (corresponding to the larynx). Because of the particular anatomy of the African penguin vocal tract and the lack of information about the actual phonation process, we modelled both the resonance in a single tube and in two tubes^70^ using a MATLAB-based computer program for vocal tract acoustic response calculation (VTAR, Vocal Tract Acoustic Response^71^). The effect of the air resonating in one or both the tracheal tubes accounted for a 8–10% variation in formant position and a 3–5% variation in formant dispersion^72^ in the contact calls and in the ecstatic display songs. The acoustic response of the vocal tract models and the visual inspection of the spectrograms indicated 5 formants below 3500 Hz for the contact calls and 5 formants below 4000 Hz for the ecstatic display songs. Finally, for each ecstatic display song, in order to describe the variation of this multi-syllable vocalisation among individuals, we firstly identified the three types of syllables described by Favaro *et al.*^45^. Further, we measured the number of syllables type 1, type 2 and type 3, the sum of the intervals type 1 (∑Type1), type 2 (∑Type2) and type 3 (∑Type3), and the total duration of the song. However, we limited the spectral analysis performed on display songs to the syllable type 2, because *f*_0_ and formants parameters were impossible to measure in the other syllable types (for more details on this methodology see also Favaro *et al.*^45^; Thumser & Ficken^46^). If the song contained more than one syllable type 2, we calculated average values for all the spectral parameters.

The acoustic measurements were carried out using a series of custom built scripts^6^,^29^,^70^ in Praat v.5.4.01^73^. We extracted the *f*_0_ contour of each vocalisation using a cross-correlation method [Sound: To Pitch (cc) command]. We used a time step of 0.01 s, a pitch floor of 150 Hz, and a pitch ceiling of 350 Hz. From each extracted *f*_0_ contour, we obtained the frequency value of *f*_0_ at the start (*f*_0_Start) and at the end (*f*_0_End) of the call; the mean (*f*_0_Mean), minimum (*f*_0_Min) and maximum (*f*_0_Max) *f*_0_ frequency values across the call. We measured the percentage of duration from the beginning of the signal to the time at which the minimum frequency (Time *f*_0_min) and the maximum frequency (Time *f*_0_max) occurs. In addition, we obtained the *f*_0_ mean absolute slope (*f*_0_AbsSlope), which is a measure of the average local variability in *f*_0_, by computing the average slope between adjacent points on the pitch curve. Furthermore, we calculated the number of complete cycles of fundamental frequency modulation per second (FMRate) and the ratio between the total FM variation and FM rate (FMExtent). We also calculated Jitter [the mean absolute difference between frequencies of consecutive *f*_0_ periods divided by the mean frequency of *f*_0_ (Jitter (local) command)] and Shimmer [the mean absolute difference between the amplitudes of consecutive *f*_0_ periods divided by the mean amplitude of *f*_0_ (Shimmer (local) command)] values. Jitter and Shimmer are measures of the cycle-to-cycle variations of fundamental frequency and amplitude, respectively. For a detailed description of the algorithms used by Praat to calculate Jitter and Shimmer, please refer to Boersma^74^. We quantified the mean harmonics-to-noise ratio value (Sonority), the number of complete cycles of amplitude modulation per second (AMRate), the mean peak-to-peak variation of each In modulation per second (AMExtent), and the mean variation per second (AmpVar). Moreover, we extracted the contour of the first four formants (F_1_–F_4_) of each call using a Linear Predictive Coding analysis (LPC; Sound: To Formant (burg) command; for contact call: time step = 0.045 s, maximum number of formants = 5, maximum formant = 3500 Hz; ecstatic display song: time step = 0.045 s, maximum number of formants = 5, maximum formant = 4000 Hz) and we calculated the average frequency values. To check if the Praat software accurately tracked the formants, the outputs of the LPC analysis were visually inspected in real time together with the spectrogram and we corrected for octave jumps when necessary. In addition, we calculated the formant dispersion (ΔF) using the methods described by Reby & McComb^29^. From the ΔF, for each vocalisation we estimated the vocal tract length of the caller using the following equation: VTL = 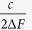
where c is the approximate speed of sound in the mammalian vocal tract (350 m/s) and the vocal tract is modelled as an uniform tube open at one end and closed at the other.

### Statistical analysis

For each acoustic parameter we calculated the Potential of Individuality Coding (PIC). The PIC assesses the ratio between within-individual variation (CV_w_) and between-individual variation (CV_b_) of an acoustic parameter using the formula: 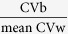 where the mean CV_w_ is the mean value of the CV_w_ for all individuals^60^. We calculated the CV_w_ using to correction for small samples (for an example, see Charrier *et al.*^15^): CV_w_ = 100 
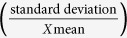
 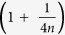 where *X*_mean_ is the mean of the sample, and *n* is the sample size for one individual. We calculated the CV_b_ according to the formula: CV_b_ = 100 
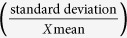
 where the standard deviation and *X*_mean_ are calculated for the total sample^75^. According to several studies^60^,^61^,^76^,^77^, acoustic parameters showing PIC > 1 have the potential to encode the individual identity information, since their intra-individual variability is smaller than their inter-individual variability.

Acoustic parameters with PIC ≥ 1.1 (Table 5) were used to perform a discriminant function analysis (DFA) using a stepwise procedure. The F-value threshold for acceptance or rejection of independent variables was set at F = 3.84. Moreover, for external validation, we used a leave-one-out cross-validation procedure. We performed two separate classifications for the contact calls and the ecstatic display songs, respectively. In both cases, the identity of the caller was used as the group identifier and the acoustic variables as discriminant variables. The percentage of classification expected by chance was calculated according to the group sizes, since the different individuals do not contributed equally to the samples. Finally, we estimate the overall significance of the DFA classification using a Yates corrected *χ^2^* test. Alpha values were set at 0.001. The analyses were performed in SPSS v.22 (IBM Corp. Released 2013. IBM SPSS Statistics for Macintosh, Version 22.0. Armonk, NY: IBM Corp.).

## Additional Information

**How to cite this article**: Favaro, L. *et al.* Vocal individuality cues in the African penguin (*Spheniscus demersus*): a source-filter theory approach. *Sci. Rep.* **5**, 17255; doi: 10.1038/srep17255 (2015).

## Supplementary Material

Supplementary Information

Supplementary Audio S1

Supplementary Video S1

## Figures and Tables

**Table 1:** Individual values of the acoustic parameters (mean ± SD) for contact calls.

Acoustic parameter	Guizzo (n = 9)	Chris (n = 14)	Baako (n = 20)	Sparrow (n = 25)	Oceano (n = 31)	Renato (n = 19)
*f*_0_Start (Hz)	277.09 ± 25.99	275.96 ± 25.99	253.66 ± 34.22	262.29 ± 21.98	248.39 ± 36.65	265.46 ± 20
*f*_0_End (Hz)	285.88 ± 25.97	291.41 ± 25.97	270.01 ± 40.47	295.79 ± 18.29	255.79 ± 35.12	281.55 ± 22.87
*f*_0_Mean (Hz)	288.6 ± 18.28	285.04 ± 18.28	256.14 ± 23.24	294.03 ± 13.63	251.62 ± 14.74	285.46 ± 16.06
*f*_0_Min (Hz)	262.52 ± 19.79	259.15 ± 19.79	230.47 ± 19.65	257.43 ± 21.04	216.72 ± 22.48	248.97 ± 12.54
*f*_0_Max (Hz)	302.31 ± 22.69	308.49 ± 22.69	287.44 ± 35.23	310.44 ± 13.01	283.8 ± 21.51	305.42 ± 13.06
Time *f*_0_Min (%)	70.2 ± 32.51	73.44 ± 32.51	66.24 ± 29.43	83.09 ± 32.77	65.56 ± 34.11	61.08 ± 42.23
Time *f*_0_Max (%)	38.88 ± 35.51	40.93 ± 35.51	39.07 ± 34.43	30.41 ± 21.8	51.92 ± 36.99	35.4 ± 28.78
*f*_0_AbsSlope (Hz/s)	134.41 ± 66.13	140.51 ± 66.13	193.26 ± 114.92	141.33 ± 43.27	219.29 ± 67.48	140.39 ± 51.89
*f*_0_Var (Hz/s)	6.52 ± 4.83	4.55 ± 4.83	3.65 ± 3.36	4.03 ± 2.35	6.69 ± 5.2	3.98 ± 2.61
FMRate (s^−1^)	1.7 ± 0.98	2.36 ± 0.98	1.97 ± 0.83	1.57 ± 0.99	2.16 ± 0.85	1.23 ± 0.63
FMExtent (Hz)	4.43 ± 2.45	2.08 ± 2.45	2.36 ± 2.32	3.27 ± 2.24	3.51 ± 2.71	3.71 ± 2.78
Jitter (%)	0.01 ± 0.02	0.04 ± 0.02	0.02 ± 0.01	0.01 ± 0.01	0.01 ± 0.01	0.01 ± 0.01
Shimmer (%)	0.16 ± 0.03	0.2 ± 0.03	0.18 ± 0.06	0.1 ± 0.06	0.11 ± 0.05	0.16 ± 0.04
Sonority	8.55 ± 3.77	4.38 ± 3.77	6.26 ± 5.21	15.98 ± 8.3	12.81 ± 4.99	8.07 ± 2.59
AMRate (s^−1^)	7.49 ± 2.28	8.23 ± 2.28	7.29 ± 1.9	5.04 ± 2.41	5.35 ± 1.8	8.44 ± 1.53
AMExtent (dB)	75.89 ± 20.41	65.89 ± 20.41	72.62 ± 23.81	146.71 ± 143.08	102.81 ± 41.06	55.85 ± 12.08
AmpVar (dB/s)	503.45 ± 28.7	502.41 ± 28.7	490.86 ± 33.99	506.66 ± 18.51	487.99 ± 22.41	457.37 ± 54.56
F_1_Mean (Hz)	572 ± 45.34	364.57 ± 45.34	487.15 ± 58.66	583.4 ± 61.58	489.61 ± 44.44	431.63 ± 104.03
F_2_Mean (Hz)	987.89 ± 48.15	888.79 ± 48.15	902.95 ± 97.19	992.24 ± 111.76	860.68 ± 49.18	940.63 ± 69.94
F_3_Mean (Hz)	1496.44 ± 102.75	1255.5 ± 102.75	1404.7 ± 108.81	1442.28 ± 187.49	1334.06 ± 94.16	1403.89 ± 114.66
F_4_Mean (Hz)	2169.33 ± 140.7	1908.71 ± 140.7	2075.4 ± 113.43	2036.96 ± 168.96	1984.42 ± 95.18	2048.11 ± 89.61
ΔF (Hz)	623.89 ± 31.43	539.75 ± 31.43	589.22 ± 34.5	595.96 ± 55.08	562.69 ± 25.83	585.95 ± 27.24
VTLest (cm)	28.14 ± 1.92	32.53 ± 1.92	29.8 ± 1.72	29.61 ± 2.76	31.16 ± 1.44	29.93 ± 1.39
Dur (s)	0.52 ± 0.12	0.57 ± 0.12	0.59 ± 0.12	0.52 ± 0.05	0.58 ± 0.07	0.79 ± 0.19

**Table 2:** Individual values of the acoustic parameters (mean ± SD) for ecstatic display songs.

Acoustic parameter	Renato (n = 10)	Picchio (n = 7)	Rico (n = 8)	Joker (n = 9)	Sky (n = 9)	Kusubiro (n = 10)	Soldato (n = 11)
*f*_0_Start (Hz)	277.41 ± 17.93	278.35 ± 20.42	287.19 ± 29.07	285.72 ± 18.73	286 ± 35.62	246.64 ± 25.48	276.67 ± 20.14
*f*_0_End (Hz)	307.49 ± 13.44	281.51 ± 27.88	302.68 ± 15.28	286.61 ± 19.82	274.14 ± 19.83	289.7 ± 14.16	290.94 ± 15.46
*f*_0_Mean (Hz)	291.66 ± 12.31	277.6 ± 9.96	283.73 ± 22.65	280.91 ± 12.37	257.23 ± 14.57	266.26 ± 9.85	260.39 ± 11.22
*f*_0_Min (Hz)	270.94 ± 14.02	245.41 ± 15.2	265.15 ± 28.57	265.26 ± 15.23	236.86 ± 17.72	238.04 ± 15.86	243.83 ± 11.73
*f*_0_Max (Hz)	309.93 ± 13.57	305 ± 16.56	313.16 ± 18.97	305 ± 12.74	306.74 ± 16.58	294.53 ± 15.08	304.44 ± 8.55
Time *f*_0_Min (%)	77.95 ± 27.4	47.85 ± 35.68	60.82 ± 38.55	72.04 ± 31.51	75.92 ± 16.11	84.26 ± 24.37	83.48 ± 15.08
Time *f*_0_Max (%)	17.38 ± 26.23	34.42 ± 23.22	43.02 ± 42.16	56.47 ± 37.46	57.88 ± 34.82	7.27 ± 11.4	34.42 ± 40.59
*f*_0_AbsSlope (Hz/s)	51.93 ± 13.08	105.14 ± 57.9	87.48 ± 54.61	63.44 ± 12.42	129.4 ± 49.31	83.94 ± 64.37	83.73 ± 29.63
*f*_0_Var (Hz/s)	1.2 ± 1.19	1.83 ± 1.67	3.06 ± 3.21	3.02 ± 3.34	3.11 ± 2.84	3.17 ± 3.39	3.17 ± 1.42
FMRate (s^−1^)	1.63 ± 0.84	2.03 ± 0.44	1.53 ± 0.4	1.93 ± 0.57	1.32 ± 0.52	1.54 ± 0.84	0.96 ± 0.52
FMExtent (Hz)	0.71 ± 0.57	0.96 ± 0.79	2.08 ± 1.95	1.53 ± 1.36	3.2 ± 4.79	2.32 ± 1.74	5.18 ± 3.84
Jitter (%)	0.006 ± 0.003	0.013 ± 0.007	0.009 ± 0.004	0.014 ± 0.009	0.009 ± 0.006	0.007 ± 0.006	0.005 ± 0.002
Shimmer (%)	0.115 ± 0.047	0.113 ± 0.044	0.127 ± 0.054	0.159 ± 0.037	0.113 ± 0.029	0.1 ± 0.067	0.078 ± 0.025
Sonority	11.742 ± 4.632	10.018 ± 4.653	9.494 ± 4.221	5.355 ± 3.736	9.276 ± 2.41	13.363 ± 4.97	12.642 ± 3.134
AMRate (s^−1^)	12.24 ± 3.12	11.13 ± 2.66	9.28 ± 2.52	11.68 ± 1.29	9.9 ± 1.54	8.89 ± 2.92	10.41 ± 1.51
AMExtent (dB)	32 ± 18.92	37.92 ± 14.82	58.05 ± 40.55	35.28 ± 9.55	45.36 ± 8.59	72.52 ± 105.66	37.07 ± 5.54
AmpVar (dB/s)	1.2 ± 1.19	1.83 ± 1.67	3.06 ± 3.21	3.02 ± 3.34	3.11 ± 2.84	3.17 ± 3.39	3.17 ± 1.42
F_1_Mean (Hz)	849.4 ± 158.5	828.7 ± 141.56	740.88 ± 97.04	713.28 ± 187.29	808.56 ± 33.72	894.3 ± 120.95	891.53 ± 104.54
F_2_Mean (Hz)	1392.15 ± 83.03	1392.57 ± 68.39	1346.13 ± 104.71	1400.07 ± 149.99	1335.87 ± 60.62	1400.7 ± 121.4	1416.14 ± 89.58
F_3_Mean (Hz)	2061.45 ± 158.42	2051.19 ± 180.34	1964.38 ± 159.62	2034.44 ± 197.75	1920.48 ± 87.79	2165.13 ± 194.26	2015.55 ± 127.33
F_4_Mean (Hz)	3090.8 ± 216.92	2966.01 ± 182.87	2799.38 ± 180.42	2896.04 ± 166.52	2926.74 ± 75.85	2979.43 ± 227.88	2836.7 ± 195.21
ΔF (Hz)	880.21 ± 56.39	857.73 ± 53.72	814.21 ± 49.16	841.86 ± 60.37	831.09 ± 23.75	875.67 ± 66.46	835.11 ± 50.47
VTLest (cm)	19.97 ± 1.29	20.49 ± 1.36	21.56 ± 1.29	20.9 ± 1.58	21.08 ± 0.6	20.1 ± 1.59	21.04 ± 1.33
Dur (s)	7.04 ± 3.03	7.59 ± 3.33	6.13 ± 1.6	6.48 ± 2.56	7.34 ± 1.38	5.94 ± 1.7	8.01 ± 2.24
Type1 (n)	8 ± 3.59	5.43 ± 2.76	7.13 ± 2.3	6.56 ± 4.69	7.44 ± 3	7 ± 2.71	6.45 ± 2.46
Type2 (n)	1.1 ± 0.32	2.57 ± 1.27	1.63 ± 0.52	1.78 ± 0.67	2 ± 0.87	1.4 ± 0.84	1.55 ± 0.69
Type3 (n)	1.8 ± 1.23	2.43 ± 1.13	3.75 ± 2.43	1.78 ± 1.09	2 ± 1.87	1.1 ± 1.6	3.18 ± 1.33
∑Type1 (s)	1.49 ± 0.61	1.02 ± 0.52	1.35 ± 0.48	1.22 ± 0.88	1.31 ± 0.59	1.38 ± 0.52	1.34 ± 0.62
∑Type2 (s)	1.6 ± 0.5	3.06 ± 1.8	1.56 ± 0.64	1.99 ± 0.82	1.69 ± 0.79	1.57 ± 1.17	1.76 ± 0.71
∑Type3 (s)	0.55 ± 0.43	0.71 ± 0.44	2.66 ± 5.02	0.44 ± 0.24	0.57 ± 0.54	0.23 ± 0.31	1.19 ± 0.36
DurType2 (s)	1.47 ± 0.37	1.14 ± 0.22	0.92 ± 0.22	1.15 ± 0.37	0.83 ± 0.06	1.07 ± 0.32	1.16 ± 0.11

**Table 3:** Name, sex, date of birth, origin, and number of vocalisations recorded for each African penguin.

Name	Sex	Hatch	Origin	Contact calls	Ecstatic displaysongs
Guizzo	F	01-12-2011	Zoom Torino, IT	9	—
Chris	M	23-12-2011	Zoom Torino, IT	14	—
Baako	M	27-10-2009	Zoom Torino, IT	20	—
Sparrow	M	24-02-2012	Zoom Torino, IT	25	—
Oceano	M	18-10-2010	Zoom Torino, IT	31	—
Renato	M	27-09-1991	Artis Zoo, NL	19	10
Picchio	M	13-11-2005	South Lake Wild Animal Park, UK	—	7
Rico	M	26-10-2006	South Lake Wild Animal Park, UK	—	8
Joker	M	28-09-1991	Artis Zoo, NL	—	9
Sky	M	28-10-2004	Bird Park Avifauna, NL	—	9
Kusubiro	M	04-11-2006	South Lake Wild Animal Park, UK	—	10
Soldato	M	18-12-2007	South Lake Wild Animal Park, UK	—	11

Contact calls (n = 118) were recorded from six penguins, while ecstatic display songs (n = 64) were recorded from seven penguins. One individual (Renato) contributed both vocalisation types.

**Table 4:** Abbreviations and descriptions for the acoustic parameters measured.

Acoustic parameter	Description
*f*_0_Start (Hz)	Frequency value of *f*_0_ at the start of the vocalisation
*f*_0_End (Hz)	Frequency value of *f*_0_ at the end of the vocalisation
*f*_0_Mean (Hz)	Mean *f*_0_ frequency value across the vocalisation
*f*_0_Min (Hz)	Minimum *f*_0_ frequency value across the vocalisation
*f*_0_Max (Hz)	Maximum *f*_0_ frequency value across the vocalisation
Time *f*_0_Min (%)	Percentage of the total call duration when *f*_0_ value is minimum
Time *f*_0_Max (%)	Percentage of the total call duration when *f*_0_ value is maximum
*f*_0_AbsSlope (Hz/s)	*f*_0_ mean absolute slope
*f*_0_Var (Hz/s)	Cumulative variation in the *f*_0_ contour in Hertz divided by call duration
FMRate (s^−1^)	Number of complete cycles of *f*_0_ modulation per second
FMExtent (Hz)	Mean peak-to-peak variation of each *f*_0_ modulation
Jitter (%)	Mean absolute difference between frequencies of consecutive *f*_0_ periods divided by the mean frequency of *f*_0_
Shimmer (%)	Mean absolute difference between the amplitudes of consecutive *f*_0_ periods divided by the mean amplitude of *f*_0_
Sonority	Harmonics-to-noise ratio
AMRate (s^−1^)	Number of complete cycles of amplitude modulation per second
AMExtent (dB)	Mean peak-to-peak variation of each amplitude modulation
AmpVar (dB/s)	Cumulative variation in amplitude divided by the total call duration
F_1_Mean (Hz)	Mean frequency values of the first formant across the vocalisation
F_2_Mean (Hz)	Mean frequency values of the second formant across the vocalisation
F_3_Mean (Hz)	Mean frequency values of the third formant across the vocalisation
F_4_Mean (Hz)	Mean frequency values of the fourth formant across the vocalisation
ΔF (Hz)	Formants spacing
VTLest (cm)	Estimated vocal tract length
Dur (s)	Total duration of the vocalisation
* Type1 (n)	Number of syllables type 1 in the song
* Type2 (n)	Number of syllables type 2 in the song
* Type3 (n)	Number of syllables type 3 in the song
* ∑Type1 (s)	Sum of the syllable type 1 intervals
* ∑Type2 (s)	Sum of the syllable type 2 intervals
* ∑Type3 (s)	Sum of the syllable type 3 intervals
* DurType2 (s)	Mean duration of the syllables type 2 in the song

^*^Parameters measured only to the ecstatic display song.

**Table 5:** Potential of Individual Coding (PIC) for the acoustic parameters measured on the contact call and ecstatic display song.

Acoustic parameter	PIC	
	Contact call	Ecstatic display song
*f*_0_Start	1.13	1.10
*f*_0_End	1.18	1.08
*f*_0_Mean	1.42	1.31
*f*_0_Min	1.39	1.23
*f*_0_Max	1.21	1.00
Time *f*_0_Min	0.97	0.92
Time *f*_0_Max	1.00	0.97
*f*_0_AbsSlope	1.15	1.23
*f*_0_Var	1.08	1.01
FMRate	1.04	1.10
FMExtent	0.98	1.30
Jitter	1.60	1.21
Shimmer	1.23	1.09
Sonority	1.14	1.04
AMRate	1.17	1.08
AMExtent	1.85	1.82
AmpVar	1.09	1.22
F_1_Mean	1.49	1.10
F_2_Mean	1.24	1.01
F_3_Mean	1.17	1.05
F_4_Mean	1.13	1.09
ΔF	1.20	1.05
VTLest	1.18	1.03
Dur	1.26	1.01
Type1	—	0.97
Type2	—	1.15
Type3	—	0.99
∑Type1	—	0.95
∑Type2	—	1.12
∑Type3	—	2.23
DurType2	—	1.32

Acoustic parameters showing PIC ≥ 1.1 are presented in bold type.
