# Supplementary material for: Vocal individuality cues in the African penguin (Spheniscus demersus): a source-filter theory approach
Source: Sci Rep. 2015 Nov 25;5:17255. doi: 10.1038/srep17255 (PMC4658557; doi:10.1038/srep17255)
Supplement: Supplementary Information [file srep17255-s1.pdf]

Vocal individuality cues in the African penguin (*Spheniscus demersus*): a source-filter theory approach

Livio Favaro[1]*, Marco Gamba[1], Chiara Alfieri[1], Daniela Pessani[1], Alan G. McElligott[2]

[1] Department of Life Sciences and Systems Biology, University of Turin, Via Accademia Albertina 13, 10123 Turin, Italy.

[2] Biological and Experimental Psychology, School of Biological and Chemical Sciences, Queen Mary University of London, Mile End Road, London E1 4NS, UK.

*E-mail: livio.favaro@unito.it

Supplementary Table

Table S1. Percentage of accuracies of the stepwise leave-one-out DFA for each penguin against those expected by chance alone. P values were obtained using a Yates corrected χ^2 test.

Contact calls						Ecstatic display songs					
Penguin	n	Expected (%)	Observed (%)	χ^2	Significance	Penguin	n	Expected (%)	Observed (%)	χ^2	Significance
Guizzo	9	7.6	33.3	19.0	P < 0.001	Renato	10	14.1	44.4	20.05	P < 0.001
Chris	14	11.9	71.4	48.8	P < 0.001	Picchio	7	15.6	70.0	41.46	P < 0.001
Baako	20	16.9	40.0	12.71	P < 0.001	Rico	8	10.9	57.1	36.52	P < 0.001
Sparrow	25	21.2	72.0	35.2	P < 0.001	Joker	9	15.6	80.0	51.00	P < 0.001
Oceano	31	26.3	80.6	35.9	P < 0.001	Sky	9	12.5	37.5	16.01	P < 0.001
Renato	19	16.1	73.7	44.2	P < 0.001	Kusubiro	10	14.1	66.7	40.75	P < 0.001
						Soldato	11	11.5	72.2	41.16	P < 0.001
Total	118	16.7	61.8	215.47	P < 0.001	Total	64	14.28	62.5	36.43	P < 0.001

Supplementary Audio and Video files

Audio S1. Audio file of a contact call of African penguin uttered to maintain cohesion with colony members located out of visual range.

Video S1. Video of an ecstatic display song of African penguin uttered during the breeding season.
